# One‐Step Synthesis of Copper Single‐Atom Nanozymes for Electrochemical Sensing Applications

**DOI:** 10.1002/smsc.202300259

**Published:** 2024-02-10

**Authors:** Guillermo Tostado‐Blazquez, Saptami Suresh Shetty, Saravanan Yuvaraja, Jose L. Cerrillo, Veerappan Mani, Khaled Nabil Salama

**Affiliations:** ^1^ Sensors Lab Advanced Membranes and Porous Materials Center (AMPMC) Computer, Electrical and Mathematical Science and Engineering (CEMSE) Division King Abdullah University of Science and Technology (KAUST) Thuwal 23955‐6900 Saudi Arabia; ^2^ KAUST Catalysis Center (KCC) King Abdullah University of Science and Technology (KAUST) Thuwal 23955‐6900 Saudi Arabia

**Keywords:** laser‐scribed graphene, nanozymes, oxidative stress, reactive oxygen species, **s**ingle‐atom catalysts

## Abstract

Single‐atom nanozymes (SANs) combine the natural enzymatic properties of nanomaterials with the atomic distribution of metallic sites over a suitable support. Unfortunately, their synthesis is complicated by some key factors, like poor metallic loading, aggregation, time consumption, and low yield. Herein, copper SANs, with a surface metal loading (1.47% ± 0.16%) are synthesized, through a green, facile, minimal solution processing, single‐step procedure, using a CO_2_ laser to promote the anchoring of the metallic precursor while simultaneously generating the laser‐scribed graphene (LSG) support out of a polyimide sheet. The presence of the atomic Cu on the LSG surface is verified using high‐angle‐annular dark‐field–scanning transmission electron microscopy and X‐ray photoelectron spectroscopy. To explore the advantages incurred by the incorporation of Cu SANs on LSG, the material is used as a working electrode on an electrochemical sensor for the amperometric detection of H_2_O_2_, achieving a detection limit of 2.40 μM. The findings suggest that CuSANs can confer enhanced sensitivity to H_2_O_2_, which is essential for oxidative stress assessment, reaching values up to 130.0 μA mM^−1^ cm^−2^.

## Graphical abstract

A single‐step procedure, using a CO_2_ laser, is developed to synthesize copper single‐atom nanozymes with a high surface metal loading, while simultaneously generating laser‐scribed graphene support out of a polyimide sheet.

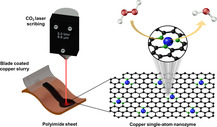

## 1 Introduction

Oxidative stress, an imbalance between the production of oxidants and defensive antioxidants, has been reported as a crucial factor for the onset and progression of several chronic or degenerative conditions like cancer, diabetes, neurological, and cardiovascular diseases.^[^ ^1^ ^]^ During cellular respiration, the reduction of molecular oxygen (O_2_) by one electron leads to the formation of some highly toxic oxidants, termed reactive oxygen species (ROS), which are the key species implicated in oxidative stress.^[^ ^2^ ^]^ Initially, O_2_ is reduced to a relatively stable intermediate, superoxide anion (O_2_•^−^), which undergoes dismutation to form hydrogen peroxide (H_2_O_2_), which is the most stable oxidant among the ROS family. Subsequently, H_2_O_2_ can be partially reduced to a hydroxyl radical (OH•) or fully reduced to water.^[^ ^3^ ^]^ These ROS can cause oxidative stress by consuming the antioxidant reserve, which can have negative ramifications for the integrity of the cellular building blocks.^[^ ^4^ ^]^ Thus, different approaches were developed to measure ROS levels, including direct measurement by electron spin resonance, electrochemical, or fluorescent sensors.^[^ ^5^ ^]^ Alternatively, indirect measurements include determination of total antioxidant capacity and measuring metabolic oxidation products.^[^ ^6^ ^]^ Within them, electrochemical sensors have been positioned as a great candidate for the assessment of oxidative stress; however, the vast majority rely on immobilized enzymes or antibodies as their biorecognition element.^[^ ^7^ ^]^ Although the utilization of these biomolecules confers specificity to the sensor, they have low operational stability and are susceptible to harsh physicochemical conditions, which make them costly to produce and store.^[^ ^8^ ^]^ To overcome the limitations of naturally derived enzymes, the development of alternative robust versions of biological selective catalysts has attracted significant attention.^[^ ^9^ ^]^ It has been reported that various nanomaterials demonstrate physicochemical properties that are similar to those of a natural enzyme receiving the name “nanozymes”. They combine the advantages of their natural counterparts with high stability, large surface area, low‐cost large‐scale production, tunable catalytic activity, and ease of modification.^[^ ^10^ ^]^

Recently, a subclass of nanozymes known as single‐atom nanozymes (SANs) has been demonstrated to further improve the catalytic activity and selectivity of nanozymes. This is possible as decreasing the size of the material enhances the creation of unsaturated coordination sites along with a surface energy increment due to the higher number of surface atoms.^[^ ^11^ ^]^ They integrate the natural enzyme‐mimicking capabilities of nanozymes to the atomical distribution of isolated metallic active sites over a substrate, derived from single‐atom catalyst technologies.^[^ ^12^ ^]^ SANs are becoming increasingly popular in biosensing applications such as colorimetric, electrochemical, photoelectrochemical, chemiluminescence, and electrochemiluminescence, as well as in biomedical applications including wound healing, antimicrobial, cancer therapy, and cytoprotection.^[^ ^13^ ^]^ A summary of the state‐of‐the‐art applications of SANs is presented in Table S1, Supporting Information. To tap into the potential of this technology, many synthetic approaches were developed to realize SANs, such as wet chemistry, pyrolysis, and atomic layer deposition.[^13^, ^14^] Unfortunately, these techniques are limited by their poor metal loading, aggregation into nanoparticles, and low yield.^[^ ^15^ ^]^ Accordingly, finding alternative techniques for SAN synthesis that overcome these obstacles is imperative. As most of these methods depend on multiple steps, are laborious, and require excessive solution processing, it is crucial to develop an alternative synthetic pathway that allows for practical scaling of SANs. However, developing a solid‐state procedure for SANs is challenging as the interaction of substrate and metallic precursor is commonly carried out in solution state for SAN formation. As the choice of support can influence anchoring and stability, its adequate material selection is crucial for the synthesis of SANs. An ideal support should promote uniform dispersion of the metal atoms, optimize its coordination environment, ensure accessibility of active sites, and prevent agglomeration.[^13^, ^16^] Carbon materials, particularly graphene, possess the right structure, porosity, morphology, and ease of functionalization to be used as such. Its 2D nature conveys high surface area, useful for uniform dispersion of the metal atoms with a high loading percentage. Its excellent electrical conductivity facilitates rapid electron transport to and from the catalytic sites. Its chemical stability over a wide potential range makes it useful for electrochemical applications.^[^ ^17^ ^]^ The fabrication of 3D porous graphene electrodes can be achieved by a simple and solution‐free process consisting of laser‐scribing thermoplastic resins, like polyimide (PI), with a CO_2_ laser.^[^ ^18^ ^]^ This method conveys some advantages like its scalability, simplicity (no postprocessing), low cost, and high resolution.^[^ ^19^ ^]^ A particular attribute of this technique is the possibility to integrate heteroatoms like nitrogen within the porous graphene lattices due to the high temperatures reached during the scribing process.

Selectively embedding single atoms of transition metals, such as copper, cobalt, iron, and nickel by promoting a metal—N—C bond could provide a low‐cost and efficient way to realize SANs.^[^ ^20^ ^]^ Additionally, it is worth noting that this particular arrangement resembles MN_x_ sites to those found on natural metalloenzymes.^[^ ^11^ ^]^ Except for two reports, laser‐scribed graphene (LSG) electrodes have not yet been combined with single‐atom catalysts.^[^ ^21^ ^]^ These two reports depend on extensive procedures including drop casting, freeze drying, and vacuum filtering graphene oxide sheets and multiple steps. To the best of our knowledge, this is the first synthetic report on the laser irradiation‐induced simultaneous synthesis of graphene and SANs in one single step.

Herein, we present a facile method for the simultaneous graphitization of a 3D porous LSG electrode and anchoring of copper single atoms via CO_2_ laser ablation. We demonstrate how this single‐step and environmentally friendly method enables the scalable realization of copper SANs (CuSANs) embedded on LSG to achieve peroxidase‐like activity. Using CuSAN as a working electrode, we showcase its capability in detecting H_2_O_2_ electrochemically. Through amperometric testing, we found that the system could achieve a limit of detection (LoD) of 2.40 μM, highlighting its great potential for the assessment of oxidative stress.

## 2 Results and Discussions

**Figure 1:**
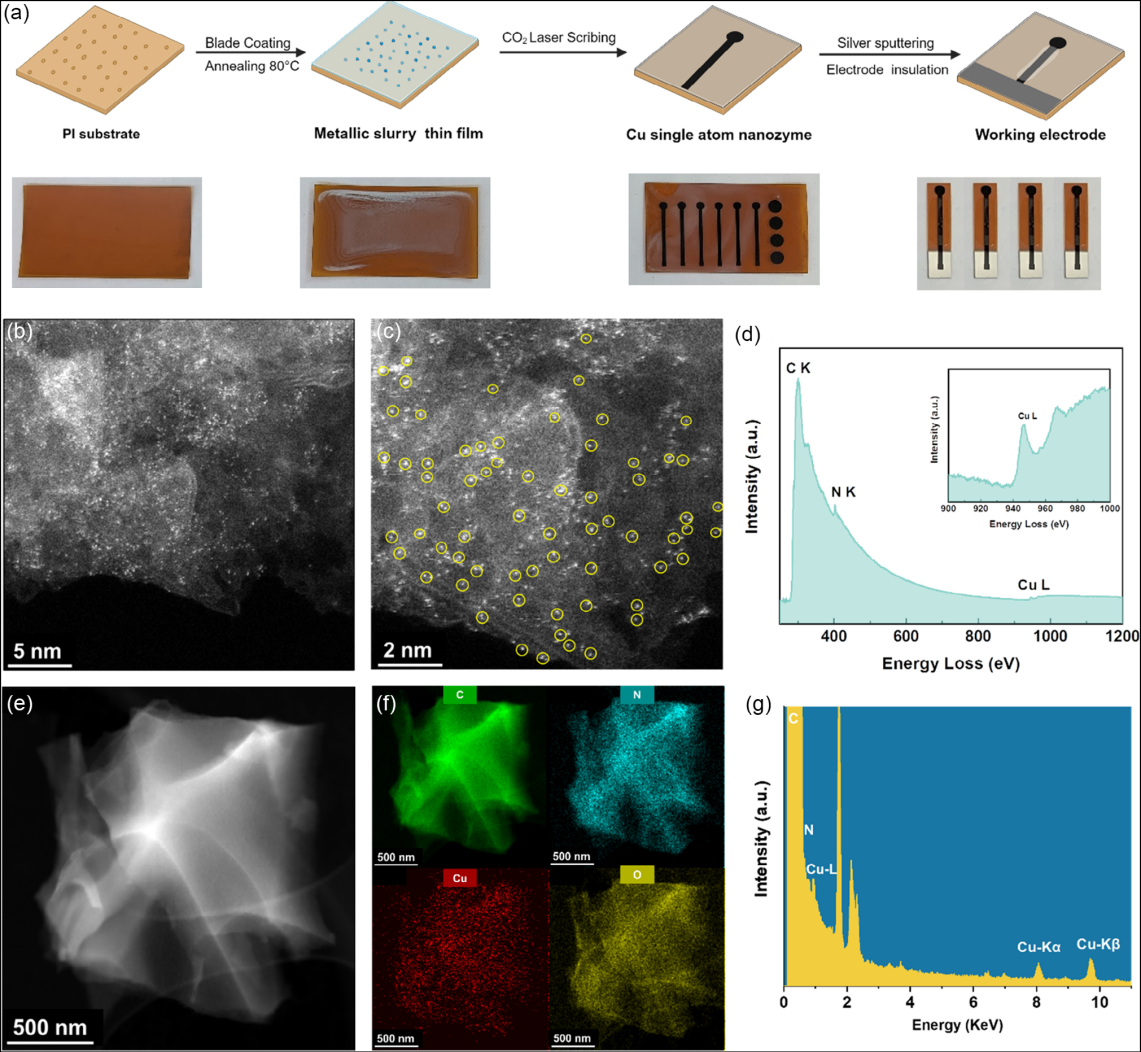
a) Schematic representation of the synthesis pathway of CuSAN. b, c) HAADF–STEM images of CuSAN at two different magnifications. The yellow circles highlight the atomic Cu single atoms. d) Core‐loss EELS spectrum of CuSAN displaying the elemental edges of the main constituting elements copper, nitrogen, and carbon. e–f) EDS elemental mapping of CuSAN and g) EDS spectra of CuSAN.

**Figure** **1**a depicts the synthesis steps optimized for preparing CuSANs and the optical images of the materials at each stage of the synthesis. Briefly, to synthesize CuSANs, a metallic slurry composed of PVA, CuNO_3_, and urea was blade‐coated onto PI sheets and annealed at 85 °C. By incorporating urea and PI as nitrogen and carbon sources, a Cu–N–C type of bond may form to emulate an enzymatic center of activity. The PVA was employed to provide some viscosity to the slurry, as an adequate thickness was imperative to be able to achieve a uniform film via blade coating. During the annealing process, the water present in the slurry evaporated leaving behind a dry film with immobilized copper atoms. Finally, the coated substrates were laser irradiated; inducing extreme temperature rises of over 2500 °C at the laser's focal spot. This process promotes the rearrangement and breakage of bonds such as C––O, C═ = O, and N––C to form graphitic bonds, accompanied by the release of gases creating a porous network of bonds. Due to the presence of Cu on the dry film, these heteroatoms are also combined within the material, resulting in their anchoring on the newly generated graphitic support.

Scanning electron microscope (SEM) images were acquired to study the surface morphology of the electrodes. To avoid the charging effect while imaging, samples were sputtered with 2 nm of gold. The SEM images of the LSG and CuSAN are presented in Figure S1, Supporting Information. As expected, LSG exhibits a characteristic porous sheet‐like morphology, which is consistent with previous reports.^[^ ^22^ ^]^ Images of the CuSAN surface also showcase a porous 3D arrangement of vertically aligned graphene layers. Additionally, the potential incorporation of copper did not adversely affect the support structure or generate visible agglomerates of copper crystals or nanoparticles. Next, high‐angle‐annular dark‐field scanning transmission electron microscopy (HAADF–STEM) was utilized to investigate the presence of copper single atoms anchored on the LSG support (Figure 1b,c). Uniformly dispersed bright dots corresponding to Cu atoms were observed due to the difference in atomic weight between Cu and the graphitic support. In addition, no evident presence of Cu nanoparticles or bigger aggregates was observed in the transmission electron microscopy (TEM) scans. The sizes of the atomic Cu particles were found to be quite close in value to that of the atomic radius of copper (128 pm), as shown in Figure S2, Supporting Information. It is worth noting that in addition to single isolated atoms, some other clusters of pairs and thirds of atoms were also found, corresponding to the higher radius values. In the core‐loss electron energy loss spectroscopy (EELS) spectrum of CuSAN, the characteristic elemental edges of copper and nitrogen over the graphene support can be observed, affirming the presence of the constituents in the sample (Figure 1d). Moreover, energy‐dispersive X‐ray spectroscopy (EDS) mapping was conducted in conjunction with STEM to demonstrate the uniform elemental distribution of C, N, O, and Cu across the sample (Figure 1e,f). Predictably, the elemental signatures of the major elements C, N, Cu, and O were observed in the EDS spectra (Figure 1g).

**Figure 2:**
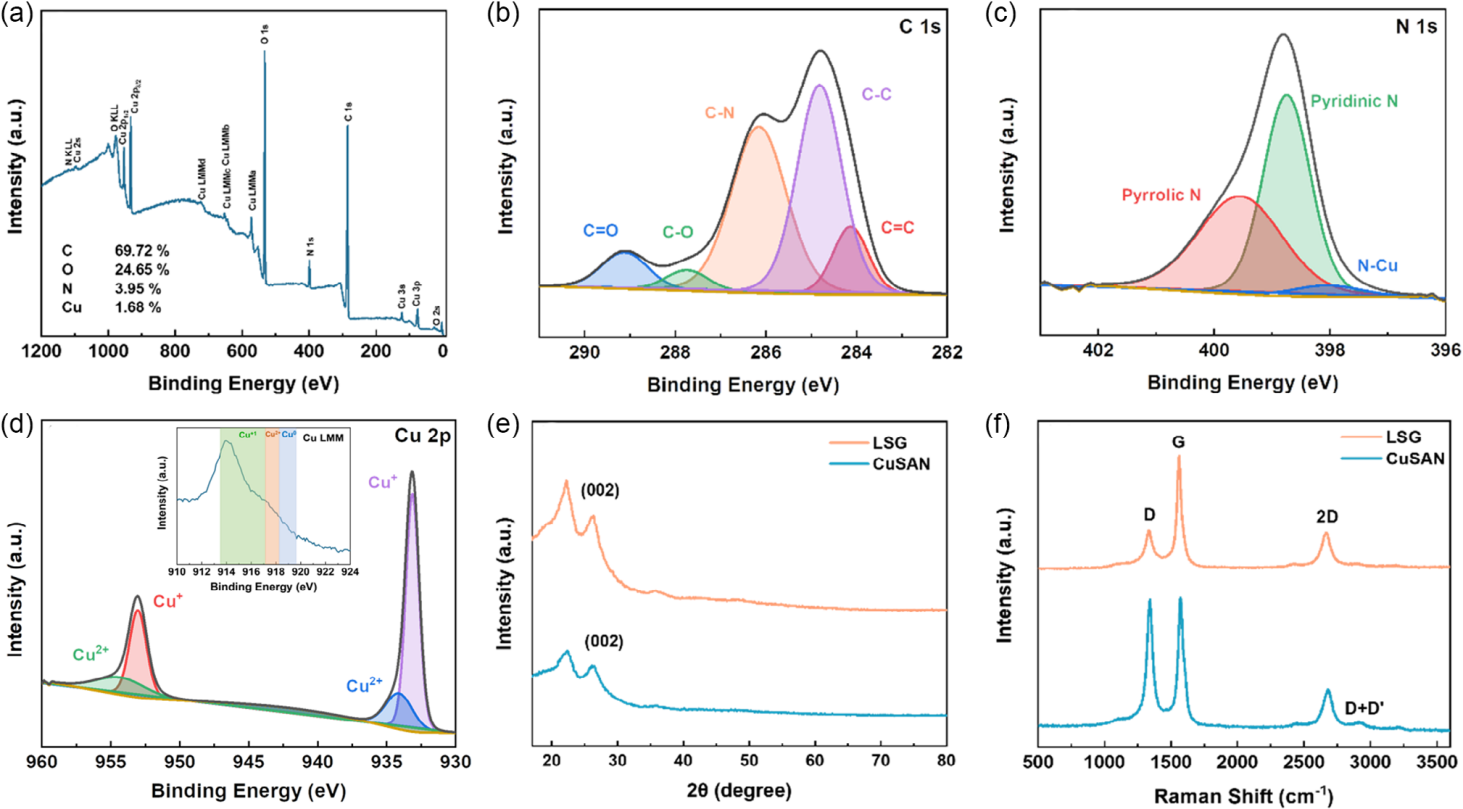
a) XPS wide spectra of the CuSAN and high‐resolution scans of b) C 1*s*, c) N 1*s*, and d) Cu 2p. Inset in 2(d): Auger spectra of the Cu 2p. e) XRD and f) Raman spectra of bare LSG and CuSAN.

X‐ray photoelectron spectroscopy (XPS) was conducted to study the surface elemental composition and chemical state of the sample (**Figure** **2**a–d **)**. From the C1*s* spectra, two intense peaks appear at 284.1 and 284.8 eV, associated with *sp* ^2^ and *sp* ^3^ hybridized carbon. The former arose from the presence of graphene, while the latter from the unconverted carbon from the PI. The presence of three more peaks, centered at 286.2, 287.8, and 289.1 eV, suggests the binding of nitrogen (C–N) and oxygen to carbon (C–O or C═O), respectively.^[^ ^23^ ^]^ The N1*s* spectrum exhibits two primary peaks at 398.7 and 399.5 eV, which are correlated to pyrrolic and pyridinic nitrogen, respectively. This is consistent with the peaks observed on the C1*s* spectra. The presence of these bonds suggests the anchoring of copper to the LSG via Cu–N–C as both types of N bonds can act as good coordination sites. This is corroborated by the presence of a small peak at 397.87, which is associated with N––Cu bond.^[^ ^24^ ^]^ Finally, two prominent peaks were observed on the Cu2*p* spectra at 933.0 and 953.0 eV, attributed to Cu2*p*_3/2_ and Cu2*p*_1/2_, respectively.^[^ ^25^ ^]^ These orbital peaks can be deconvoluted into four separate peaks, suggesting the presence of Cu^2+^ and Cu^+^/C^0^. Solely from XPS data, it is hard to discern between Cu^+^ and metallic Cu; therefore, evaluation of the copper L‐inner level‐M‐inner level‐M‐inner level electron transition Auger spectra was required to claim the presence of one species or the other. As shown in the inset of Figure 2d, there is a characteristic peak at 914.0 eV, attributed to the presence of Cu^+^ confirming the presence of both oxidation states.^[^ ^26^ ^]^ Furthermore, based on the area under the curve from each peak corresponding to Cu^+^ and Cu^2+^, the percentage of each species was determined to be 74% and 26%, respectively. Finally, surface copper loading was determined to be 1.47% ± 0.16% from the average of three different samples (Figure S3, Supporting Information).

Inductively coupled plasma–optical emission spectroscopy (ICP‐OES) was conducted, as reported in the Experimental Section, to determine the bulk copper loading percentage of the sample. In Table S2, Supporting Information, we report the obtained values, which represent the average of five replicates of each measurement at six diverse wavelengths associated with copper. A total average value yielded 0.22 ± 0.04 wt%. These results demonstrate that the blade coating method employed for the thin‐film processing of the metallic precursor; therefore, the copper loading, is consistent and reproducible.

X‐ray diffraction (XRD) was conducted to analyze the crystallinity of the material and rule out the presence of Cu aggregates, which could generate crystal peaks. As shown in Figure 2e, the XRD spectra for bare LSG and the CuSAN are almost identical. Two prominent peaks were observed at 2*θ*% = ≈22.2° and ≈26.3°. These peaks are attributed to the polyimide sheet and the (002) plane, originating from the high degree of graphitization, which is consistent with previous reports.^[^ ^25^, ^27^ ^]^ Additionally, the lack of copper crystal peaks, associated with the presence of copper nanoparticles, expected at 2*θ* = 43°, 50°, and 74°, assigned to the (111), (200), and (220) planes of a pristine copper face‐centered cubic structure, confirms the absence of agglomerated or crystalline state copper.^[^ ^28^ ^]^ To obtain additional insight into the carbon bond structure, the samples were analyzed via Raman Spectroscopy. Three main peaks were observed, as shown in Figure 2f, D peak at ≈1,350 cm^−1^, G peak at ≈1580 cm^−1^, and 2D peak at ≈2680 cm^−1^. In the CuSAN spectra, the intensity of the D peak is equal to that of the G peak, which indicates a high level of defects or doping. Conversely, the intensity of the D peak appears to be less than that of the G peak on the undoped LSG. Furthermore, the low‐intense and broad 2D peak observed for both samples confirms the multilayered arrangement as previous reports show that a single‐layer graphene sheet presents a prominent and sharp 2D peak, almost two times more intense than the G peak. Finally, the presence of the D + D’ peak at ≈2940 cm^−1^ in the CuSAN spectra confirms doping by copper as the peak requires the defects to be active.^[^ ^29^ ^]^

To understand the effect of the H_2_O_2_ analyte on the surface of CuSAN, we have used Kelvin probe force microscopy (KPFM) analysis.^[^ ^30^ ^]^ From KPFM analysis, we can determine the contact potential difference (CPD) that represents the concentration of charge carriers.^[^ ^31^ ^]^ By measuring the CPD value of the sensor before and after reacting with the target analyte, crucial information about the reduction reaction is concluded.^[^ ^32^ ^]^ These obtained CPD values can be further used to extract the work function (*ϕ*) of the materials under test using Equation (1).

(1) $$\text{CPD}\left(V\right)=\Phi_{\text{TIP}}-\Phi_{\text{SAMPLE}}$$

Here, *ϕ*_TIP_ = the work function of the KPFM tip used to measure the CPD of the materials, *ϕ*_SAMPLE_ = the work function of the sample under test, and CPD (*V*) = the CPD values obtained from KPFM maps. In the KPFM analysis, the first step is to obtain *ϕ*_TIP_ using a standard highly ordered pyrolytic graphite (HOPG) sample. For doing KPFM mapping, we used the standard FMV‐PT tip, having Pt/Ir protective coating on the front and backside of the tip, procured from Bruker. It is necessary to know the values of both CPD (*V*) and *ϕ*_SAMPLE_ to find *ϕ*_TIP_ using Equation (1). We used the standard HOPG sample to obtain *ϕ*_SAMPLE_, its work function is around 4.6 eV, obtained from the literature. The FMV‐PT tip is used to scan the surface of HOPG (Figure S4a, Supporting Information) to obtain potential mapping (Figure S4b, Supporting Information). The mean CPD value of the HOPG sample is 263.5 mV. Both CPD (263.5 mV) and *ϕ*_SAMPLE_ (4.6 eV) values were used in Equation (1) to obtain the work function of the tip (*ϕ*_TIP_) which is 4.86 eV. The second step is to find the work function of the CuSAN sample (*ϕ*_1_).

(2) $$\Phi_{1}=\Phi_{\text{TIP}}-{\text{CDP}}_{1}$$

**Figure 3:**
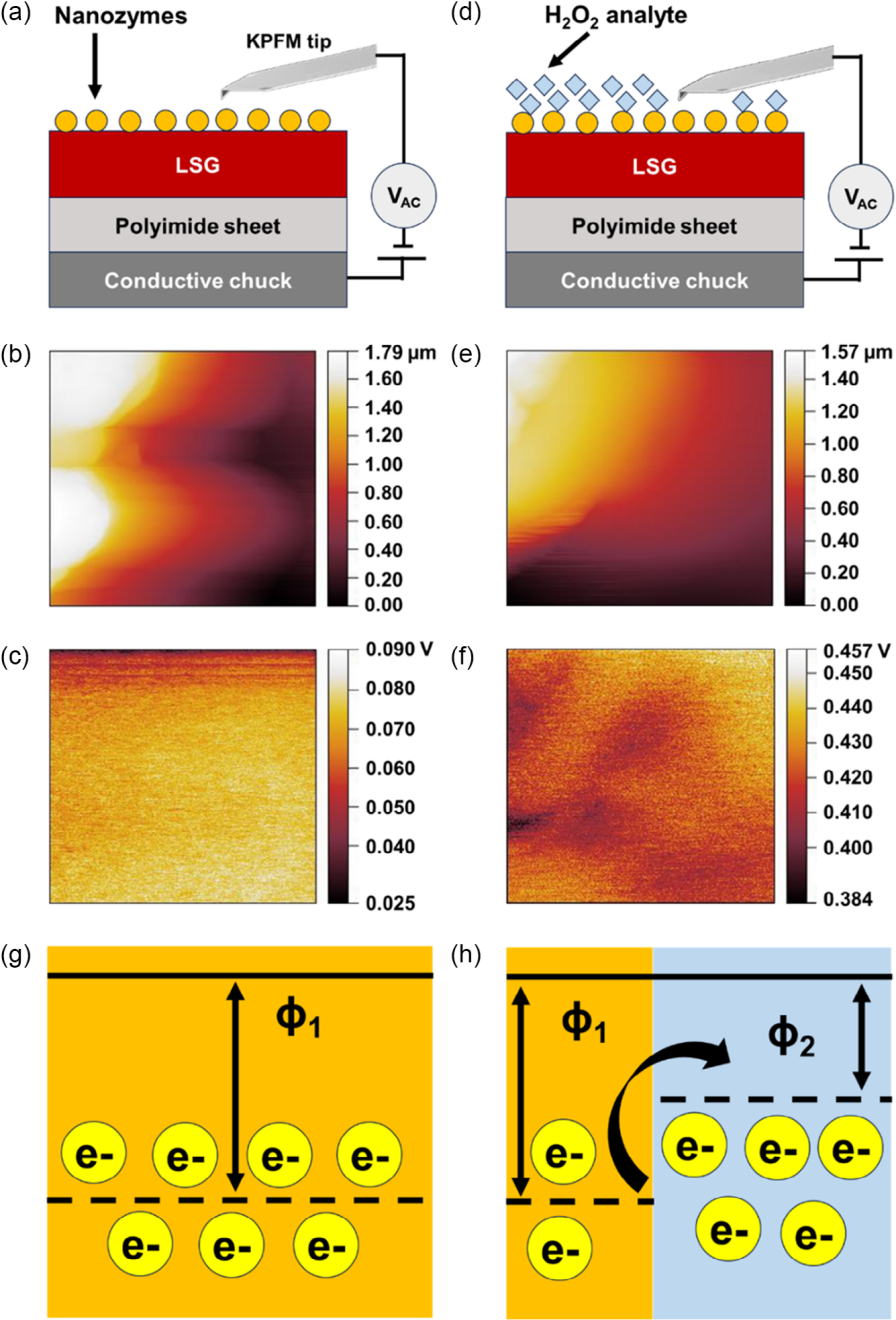
a) Experimental setup, b) surface, and c) KPFM mapping of the CuSAN nanozyme sample before reacting with H_2_O_2_. d) Experimental setup, e) surface, and f) KPFM mapping of the CuSAN nanozyme sample after reacting with H_2_O_2_. The work function plot of the CuSAN nanozyme sample g) before and h) after H_2_O_2_ analyte interaction.

Here, *ϕ*_1_ = the work function of the CuSAN sample under test. CPD_1_ (*V*) = the mean CPD value of the CuSAN sample. In this step, we are set to calculate the work function (*ϕ*_1_) of the CuSAN sample under test using Equation (2). We have performed KPFM mapping of the sample under tests to obtain CPD_1_ (**Figure** **3**a). The surface and KPFM mapping of the sample under test is shown in Figure 3b,c. The mean CPD value (CPD_1_) of the sample under the test is 70 mV. Using the CPD_1_ and previously obtained *ϕ*_TIP_ values in Equation (3), the calculated value *ϕ*_1_ is around 4.79 eV. The third step is to find the work function of the CuSAN after its electrocatalytic reaction with H_2_O_2_ (*ϕ*_2_), using Equation (3).

(3) $$\Phi_{2}=\Phi_{\text{TIP}}-{\text{CDP}}_{2}$$

Here, *ϕ*_2_ = the work function of the CuSAN sample after being treated with H_2_O_2_. CPD_2_ (*V*) = the mean CPD value of the CuSAN sample after being treated with H_2_O_2_. Here, we present the method used to calculate the work function of the CuSAN under tests after introducing the H_2_O_2_ analyte (*ϕ*_2_) via Equation (3). The setup used to do KPFM analysis of the sample under tests is shown in Figure 3d. The surface and KPFM mapping of the sample under test is shown in Figure 3e,f. The CPD_2_ value of the sample under test is around 422 mV obtained from the KPFM mapping (Figure 3f). Both CPD_2_ and *ϕ*_TIP_ values were used in equation (3) to obtain the *ϕ*_2_ around 4.44 eV. Finally, the values of *ϕ*_1_ and *ϕ*_2_ are compared and analyzed. The *ϕ*_1_ and *ϕ*_2_ values are 4.79 and 4.44 eV, respectively. Figure 3g,h showcases the change in the work function of the CuSAN sample after its electrocatalytic reaction with the H_2_O_2_. Notably, the work function of the CuSAN sample (*ϕ*_2_) after reacting with H_2_O_2_ is ≈ 0.352 eV less than its *ϕ*_1_ counterpart. The decrease in the work function is attributed to the intake of more electrons by H_2_O_2_ from the CuSAN surface. The decrease in the work function and increase in electrons confirm the drastic improvement in reduction current after introducing H_2_O_2_.

### 2.1 Electrochemical Properties of CuSAN

**Figure 4:**
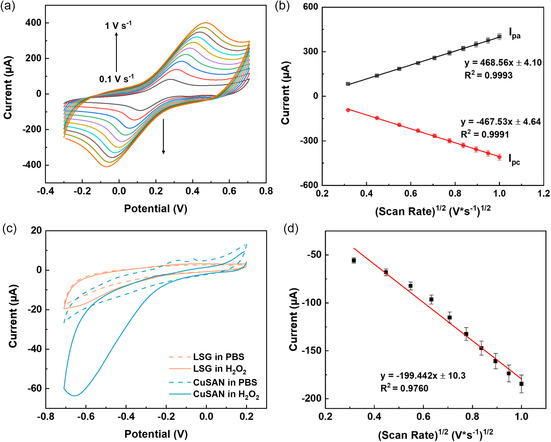
a) CV responses of CuSAN electrode at different scan rates, for 5.0 mM K_3_Fe(CN)_6_ suspended in 0.1 M KCl where each curve represents a 0.1 V s^−1^ increment and b) corresponding calibration plot between response current versus square root of scan rate. c) CV responses of CuSAN and unmodified LSG electrode to 1.0 mM H_2_O_2_ at a scan rate of 50 mV s^−1^. d) Plot between current and square root of scan rate for CV responses of CuSAN electrode to 1.0 mM H_2_O_2_ at different scan rates ranging from 0.1 to 1.0 V s^−1^.

The cyclic voltammetry (CV) response of the CuSAN electrode was investigated to understand its electrochemical properties (Figure S5a, Supporting Information). The CV shows a reduction signal at −0.37 V, which can be assigned to the oxygen reduction reaction, in addition to a couple of small humps at −0.059 and +0.030 V and the onset of a peak at +0.06 V, which are attributed to the presence of copper in the SAN.^[^ ^33^ ^]^ Next, the CV of the CuSAN electrode was tested at different scan rates, from 100 to 1000 mV s^−1^ in phosphate‐buffered saline (PBS) buffer (Figure S5b, Supporting Information). Mostly background was noticed, plus a couple of shoulder peaks which can be attributed to the residual oxygen functionalities residing on the graphene sheet surface. The peak currents are linearly increased along with the scan rate, which is a typical behavior of a surface‐confined redox process. The electrochemical behavior of the CuSAN electrode was also tested in the presence of 5.0 mM K_3_Fe(CN)_6_ dissolved in 0.1 M HCl. Different scan rates were applied between 100 and 1000 mV s^−1^ and, as shown in **Figure** **4**a,a distinct redox couple was observed in the voltammogram, matching with the characteristic redox signature of K_3_Fe(CN)_6_. At 100 mV s^−1^, the peak‐to‐peak separation value was 0.175 V, and the anodic (*I*_pe_) and cathodic peak currents were 82.89 and −92.59 μA, respectively. The response currents were linearly correlated with the square root of the scan rate for both *i*_PAQ_ and *I*_pc_ and the corresponding regression equations are given in Figure 4b. The obtained slope values of anodic and cathodic segments were averaged and utilized in the Randles–Sevcik Equation (4) to estimate the electrochemically active surface area (ECSA) of the CuSAN‐LSG electrode.

(4) $$I_{p}=2.69\times 10^{5}*n^{3/2}*A*D^{1/2}*C*v^{1/2}$$

where *I*_p_ is peak current, A is active surface area of the electrode, *D* is the diffusion coefficient (*D* = 7.3 × 10^−6^ cm^2^ s^−1^), *C* = is the concentration of [Fe(CN)_6_]^3−^ (*C* = 5 × 10^−6^ mol cm^−3^), and ν = scan rate (0.05 V s^−1^). The ECSA was found to be 0.1288 cm^2^, which represents a 61.42% increase over that of the bare LSG (Figure S6, Supporting Information).

The performance of the electrocatalytic activity of the CuSAN in reducing H_2_O_2_ was tested in the presence of 1.0 mM H_2_O_2_ solution in 10 mM PBS. Deoxygenation was done to avoid false positive results from oxygen signals. In the absence of H_2_O_2_, the baseline current of CuSAN was nearly identical to that measured in PBS. However, a clear, sharp cathodic peak was observed in the presence of H_2_O_2_. The onset potential and peak potential of the cathodic peak were determined to be −0.20 and −0.65 V, respectively. Compared to bare LSG, there is a threefold increase in current, which can be attributed to the enhanced activity of the nanozyme modification (Figure 4c). Furthermore, relative to CuSAN, the onset potential was shifted by −0.30 to −0.50 V for the bare LSG. This means that the 300 mV has less anodic potential at CuSAN compared to the LSG electrode, which indicates that CuSAN acts as a catalyst for the H_2_O_2_ reduction process, facilitating the reduction reaction in a favorable direction. Additionally, by testing the electrode at different scan rates, from 100 to 1000 mV s^−1^, a linear trend was observed between the square root of the scan rate and the current response with an *R* ^2^ value of 0.9760, suggesting a diffusion‐controlled process (Figure 4d and Figure S7, Supporting Information). The electrochemical results are consistent with the KPFM results in the previous section.

### 2.2 Electrochemical Determination of H_*2*_ *O*_*2*_

**Figure 5:**
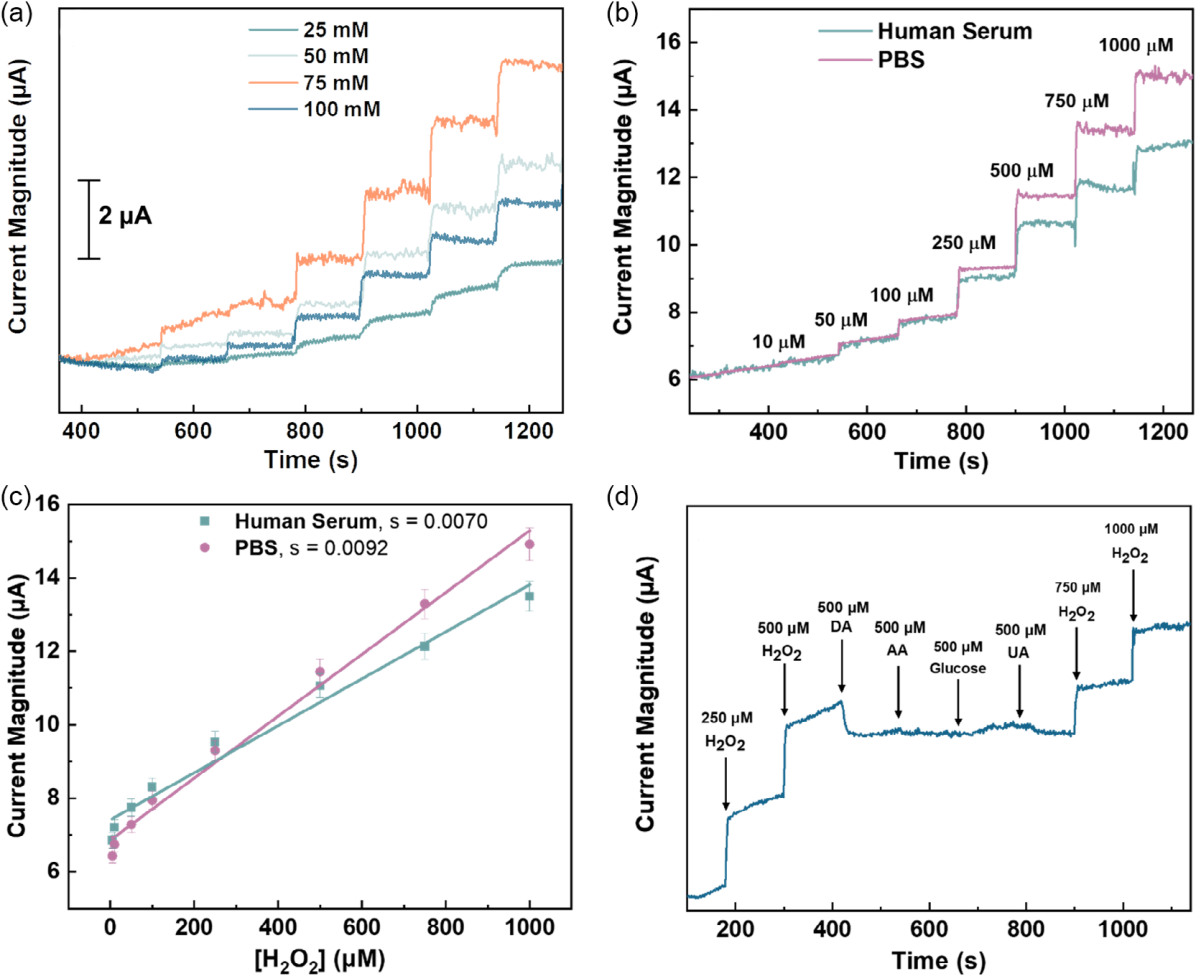
a) Amperometric signals obtained with different concentrations of copper precursor solution during the synthesis of the CuSAN: 25, 50, 75, and 100 mM. The potential was fixed at −0.38 V, the supporting electrode was PBS, pH 7.40, and each step represents the addition of an H_2_O_2_ aliquot. b) Comparison of amperometric signals of the CuSAN in PBS and artificial human serum (10%) to the addition of H_2_O_2_ aliquots (electrode potential = −0.38 V). c) Calibration curve of CuSAN behavior on artificial human serum and PBS. d) Amperometric signal of the CuSAN obtained with the addition of H_2_O_2_ and DA, AA, glucose, and UA (electrode potential = −0.38 V).

The loading content of copper on PI was optimized to provide sensitive detection of H_2_O_2_. To do so, the current–time response of the CuSAN electrode was tested with diverse versions of the material, where a different concentration of copper nitrate solution was employed for their production. The potential was fixed at −0.38 V, the supporting electrode was PBS, and the pH was 7.40. As shown in **Figure** **5**a and Figure S8, Supporting Information, a good response to the addition of H_2_O_2_ aliquots was observed in all of them by the clear step shapes. The sensitivity of each electrode was calculated by dividing the slope of their corresponding calibration curve over the geometrical area of the electrode (0.071 cm^2^). The H_2_O_2_ detection sensitivity values are estimated to be 39.89, 73.14, 103.70, and 62.39 μA mM^−1^ cm^−2^, respectively, when copper concentrations of 25, 50, 75, and 100 mM were utilized to produce CuSAN electrodes. The material processed with 75 mM copper nitrate yielded the best results. Its outstanding behavior could be attributed to reaching adequate metal loading for single‐atom formation without surpassing the threshold to form aggregates. All the CuSAN electrochemical tests present in this section are reported for CuSAN electrodes prepared with 75 mM copper nitrate precursor.

Next, amperometry experiments were performed to develop a sensitive H_2_O_2_ sensor. Different concentrations of H_2_O_2_ were tested with the CuSAN electrode, their amperogram were recorded and analyzed. Well‐defined and prompt signals are observed for each H_2_O_2_ spike (Figure 5b, pink colored line). A linear increase in the current signal response was observed when the concentration of H_2_O_2_ was increased. It was also found that the system performed over a linear range from 3.0 to 910 μM with a normalized sensitivity of 130.0 μA mM^−1^ cm^−2^ (Figure 5c, pink colored line). The limit of blank (LoB) and LoD were found to be 2.087 and 2.403 μM respectively. The analytical parameters of the CuSAN are compared with the recently reported H_2_O_2_ sensors (Table S3, Supporting Information) and with some reported Cu‐based catalysts for H_2_O_2_ reduction (Table S4, Supporting Information).[^3^, ^34^] As shown in both tables, our sensor's sensitivity surpasses most of the reported copper‐based H_2_O_2_ sensors. While they require extensive multistep laborious synthetic protocols, our catalyst is a green, minimal solution processing, and single‐step procedure. The LoD value suggests the great potential of the material to be employed for sensing H_2_O_2_ in blood, since according to literature, this biomarker can be found in ranges from 1.0 to 5 μM in healthy patients or be present from 30 to 50 μM in patients with specific diseases or chronic inflammation.^[^ ^35^ ^]^ The CuSAN sensor shows comparable performance with existing H_2_O_2_ sensors, especially showing impressive linear range and high sensitivity. The linear range continues until 910 μM, which is practically useful to determine H_2_O_2_ beyond biological H_2_O_2_. For example, this range can be useful to analyze H_2_O_2_ in samples such as bleaching and disinfectants because the H_2_O_2_ levels are at higher end in such samples. In other words, the CuSAN sensor can be used not only for practical samples with micromolar levels of H_2_O_2_ but also with samples with millimolar levels.

The practical feasibility of the CuSAN electrode as a real‐world H_2_O_2_ sensor was studied with amperometric time‐current measurements on artificial human serum by spiking it with H_2_O_2_ aliquots (1 to 1 mM) using the same CuSAN electrode (Figure 5b, cyan‐colored line). Consistent steps were observed just like in the case of spiked PBS. A comparison of the sensitivity values was conducted by determining the slope of their calibration curves and normalizing them over the geometrical surface area of the electrode (Figure 5c, cyan‐colored line). The normalized sensitivity on human serum was found to be 98.87 μA mM^−1^ cm^−2^, showcasing a 23.94% sensitivity loss in comparison with the measurements conducted on PBS, which is acceptable considering the complex components of the human serum as a matrix.

The selectivity of the sensor toward H_2_O_2_ detection was investigated in the presence of common interfering agents such as ascorbic acid, dopamine, glucose, and uric acid by chronoamperometry. The concentration range of interfering substances is chosen based on their physiological levels in human serum or blood. As shown in Figure 5d, the only occasion where a significant drop in current magnitude was recorded was with the addition of dopamine, whereas for the rest of the interferents, there was no evident interference on the amperogram. This could be attributed to the high concentration of dopamine that was initially added. Interestingly, the electrode still responded to the further addition of H_2_O_2_ with all the interferents in the medium. To corroborate if this phenomenon occurred due to the elevated concentration of interferents, which was standardized to 500 μM, a separate study was conducted for each of the interferents where their usual concentration in blood was considered as the final concentration divided over four aliquots (Figure S9, Supporting Information). Results suggest that the amount of dopamine employed in the first experiment was fouling the electrode interface, since once it was conducted with its biological concentration, no decrease in the current magnitude was observed. The rest of the interferents followed the same behavior except for glucose, which showed a small decrease at 250 μM. This effect was not observable in the first experiment due to dopamine interference at excessive concentrations. The reproducibility of the sensor was studied by measuring the current‐response signals of ten different electrodes (Figure S10a, Supporting Information). The five electrodes with a closer current value to the average (−12.697 μA) were plotted on a bar chart and their standard deviation was calculated to be 2.064 (Figure S10b, Supporting Information**)**. This value was employed to obtain the relative standard deviation, which was found to be 16.25%, which indicates fairly reproducibility of the sensor. It's worth mentioning that the reproducibility depends on the quality of graphene sheets. Electrochemical impedance spectroscopy (EIS) (Figure S11a,b, Supporting Information) was conducted to determine the reproducibility of the electrodes. The variance in charge transfer resistance (*R*_ct_) was extracted from the Bode and Nyquist plots and represented as a box plot to justify the statistical discrimination of half of the values (Figure S11c, Supporting Information). From the ICP–OES and XPS measurements, it was proved that copper loading from sample to sample is not that significantly different; therefore, we attribute the lack of reproducibility of the nanozyme to the LSG batch‐to‐batch variation. Finally, the electrochemical stability of the electrodes was studied by acquiring their CV response every seven days over a period of 28 days in the presence of N_2_‐saturated supporting PBS electrolyte, pH 7.0, with a potential range from +0.20 to −0.70 V (Figure S12, Supporting Information). The average value of the current was determined to be 29.18 μA with a standard deviation of 1.61. The shelf‐life stability was obtained by ratioing the current values obtained on the first and last day, yielding a result of 87.87%.

## 3 Conclusion

We have developed a single‐step environmentally friendly CO_2_ laser‐scribing procedure to synthesize copper SANs on graphene sheets. Unlike previously reported synthesis methods, our approach is simple, fast and conveys the simultaneous generation of the support and the anchoring of the metallic atoms. The material was characterized with morphological, spectral, and elemental techniques, confirming the successful anchoring of the copper atoms onto the LSG substrate and the absence of copper aggregates or nanoparticles, achieving an outstanding 1.47% ± 0.16% surface metal loading. We tested the electrochemical behavior of the material, using it as a working electrode through amperometric and voltammetric methods. The material showcased good performance detecting H_2_O_2_ on a micromolar range, with LoD of 2.4 μM over a linear range of 3.0 μM–1.0 mM. The simple synthesis procedure of the material and its detection performance could prove useful for the design of a point‐of‐care device for H_2_O_2_ detection in blood. The reported single‐step procedure likely can be extended for other metal atoms.

## 4 Experimental Section

4.1

4.1.1

##### Materials

Copper (III) nitrate hemi(pentahydrate), 98% (CuNO_3_)_2_ •2.5 H_2_O, was purchased from Alfa Aesar. Polyvinyl alcohol (PVA), hydrogen peroxide solution (30 wt%), dopamine hydrochloride (DA), D‐glucose, and artificial human serum were purchased from Sigma Aldrich. Uric acid (UA), L‐ascorbic acid (AA), potassium ferricyanide (K_3_[Fe(CN)_6_), and potassium chloride (KCl) were purchased from MP Biomedicals. PBS tablets containing 0.0027 M KCl and 0.137 M sodium chloride were acquired from Fisher Scientific. Commercial PI sheets (Kapton Width: 12”) were purchased from Utech Products. Ultrapure water (resistivity: 18.2 MΩ*cm^−1^ at 25 °C) from a GenPurePro UV integral water purification system (ThermoScientific) was used in all aqueous solution experiments. All the chemicals were of analytical grade and used as received without any prior treatment. Stock solutions were freshly prepared prior to their employment: 200 mM copper nitrate, 0.1 M PBS (pH 7.4), 5.0 mM K_3_Fe(CN)_6_ in 0.1 M KCl solution, 100 mM DA, 100 mM AA, 100 mM UA, and 100 mM glucose.

##### Instrumentation

Universal Laser Systems (PLS6.75) was used to pattern LSG nanosheets on a commercial PI sheet (Kapton Width: 12”, Utech Products, USA). XPS measurements were carried out in a Kratos Axis Supra DLD spectrometer equipped with a monochromatic Al Kα X‐ray source (*hν* = 1486.6 eV) operating at 75 W under a vacuum of 1 × 10^−8^ mbar, a multichannel plate, and a delay line detector. XPS survey spectra were collected at fixed‐analyzer pass energies of 160 eV and quantified using empirically derived relative sensitivity factors provided by Kratos analytical. Zeiss Merlin and TeneoVS field‐emission SEMs were used for SEM examinations. HAADF–STEM imaging, EELS, and EDS were performed using a Thermo Fisher Scientific Titan Cubed TEM (80–300 keV), equipped with a Probe Cs corrector, a high‐brightness electron gun (x‐FEG), and four in‐column 4 SDD Super‐X detectors. The probe semiconvergence angle was tuned for 24 mrad (milliradian) with a beam current of 56 pA. HAADF images were acquired using a Fischione ADF detector, with the collection semiangles of 50 (inner) and 200 mrad (outer). For measurements, the CuSANs were scratched from the PI sheet with a blade after laser irradiation, cleaned with ethanol, were drop cast on a carbon‐coated grid (300 mesh size), and dried overnight. X‐ray diffractometer (Bruker Corporation, D8 ADVANCE, and Karlsruhe, Germany) with Cu K*α* radiation (1.5406 Å) was used to record XRD data in a 2*θ* range of 5°–80°. Raman spectra analysis was performed using an Alpha3000 Apyron Witec Raman spectrometer with 532.163 nm laser source excitation at room temperature (Horiba Scientific).

KPFM was performed using the Bruker Dimension Icon Atomic force microscopy (AFM) system. For AFM topography studies, integral gain, scan rate, drive frequency, and amplitude set point were optimized at 2.69, 0.996 Hz, 5.000, 61.3 kHz, and 803.8 pm, respectively. The work function of the tip used for KPFM was measured and calibrated using the standard HOPG substrate. PalmSens4 (PS41904024817) and MUX8‐R2 Multiplexer (MUX8R21904005703) controlled by MultiTrace 4.5 software were used to perform the electrochemical experiments.

ICP–OES (5100 ICP‐OES Agilent instrument) was conducted to determine the chemical composition. Powder samples were scratched from the PI substrate and 4–8 mg were set for digestion on an UltraWAVE instrument (Milestone) at 240 °C and 35 bar for 30 min, using an acid solution containing 3.0 mL HCl + 1.0 mL HNO_3_ + 1.0 mL HF. Later, 5.0 mL of the digested sample was placed on a falcon tube with 5.0 more mL of HNO_3_. Digested samples were measured by quintupled for each of the six selected Cu emission wavelengths. Finally, 1.0 mL of each evaluated sample was further diluted with 9.0 mL of HNO_3_ and measured once more by quintupled. Standard solutions with different concentrations of Cu were previously prepared, obtaining proper calibration curves with *R* ^2^ > 0.999. Moreover, laboratory reagent blank, laboratory fortified blank, and quality control sample (QCS) were evaluated to validate the results.

##### Fabrication of CuSANs

A metallic slurry was prepared by ultrasonicating, at a frequency of 45 kHz, 5 mL of polyvinyl alcohol (PVA) in water (15% wt.), 5 mL of CuNO_3_ solution (150 mM), and 1.0 g of urea for 10 min. Simultaneously, polyimide (PI) sheets (6.3 × 3.5 cm) were ultrasonically cleaned with acetone for 10 min and subjected to oxygen plasma treatment for 10 min. The as‐prepared solution (1.0 mL per substrate) was blade coated (1000 μm height) onto the precleaned PI sheets at a 20 mm s^−1^ speed. Then, the coated substrates were annealed at 80 °C for ≈15 min or until a dry film was observed. A CO_2_ laser was used to scribe circular working electrodes with 3 mm diameter on the metallic slurry‐coated sheets. The parameters were set as follows: 2.8 W power, 4.5 cm s^−1^ speed, 1000 pulses per inch, and 1.0 mm *Z* distance. To reduce contact resistance with the utilized screen‐printed electrode connectors (SPE connector, 4 mm banana), the contacts were sputtered with a layer of 200 nm of silver. To serve as control, bare LSG electrodes were prepared by ultrasonically cleaning the PI sheets and then subjecting them directly to the CO_2_ laser according to the aforementioned parameters.

##### Electrochemical Experimental Procedure

All electrochemical measurements were carried out using deoxygenated 0.1 M PBS (pH 7.40) as the supporting electrolyte at ambient conditions, unless otherwise stated. A three‐electrode system consisting of bare LSG or CuSAN as the working electrode, a platinum wire as the counter electrode, and a silver/silver chloride (Ag/AgCl) as the reference electrode was utilized. The electrochemical cell volume was 20 mL for all the experiments. For the ECSA measurements, 5.0 mM K_3_Fe(CN)_6_ in 0.1 M KCl was used as a model redox probe system. CV tests were conducted between +0.70 and −0.30 V at different scan rates between 100 and 1000 mV s^−1^. Subsequent CV tests were conducted at different potential windows depending on the experimental goal. The chronoamperometry tests were conducted at a fixed potential of −0.38 V and the sampling interval for each subsequent aliquot was 2 min unless otherwise specified in the figure captions. A solution of 10% v/v human serum in 0.1 M PBS (pH 7.40) was spiked with the analyte of interest to conduct tests that emulated the relevant clinical conditions. EIS was conducted with a sinusoidal excitation signal with an amplitude of 0.01 V overlain on a DC applied potential of +0.28 V, which is dictated by the formal potential needed to oxidize K_3_Fe(CN)_6_. The frequency of the excitation signal was modulated from 0.1 Hz to 100 KHz with 75 frequencies interspaced on a logarithmic scale. To achieve stable readings, all EIS measurements were conducted relative to the open‐circuit potential, which was determined by applying a 0 V condition and measuring the voltage between the electrodes.

## Conflict of Interest

The authors declare no conflict of interest.

## Supporting information

Supplementary Material

## Data Availability

The data that support the findings of this study are available from the corresponding author upon reasonable request.
