# Supplementary material for: One‐Step Synthesis of Copper Single‐Atom Nanozymes for Electrochemical Sensing Applications
Source: Small Sci. 2024 Feb 10;4(4):2300259. doi: 10.1002/smsc.202300259 (PMC11935134; doi:10.1002/smsc.202300259)
Supplement: Supplementary file 1 — Supplementary Material [file SMSC-4-2300259-s001.pdf]

Supporting Information

ONE STEP SYNTHESIS OF COPPER SINGLE ATOM NANOZYMES FOR ELECTROCHEMICAL SENSING APPLICATIONS

Guillermo Tostado-Blazquez, Saptami Suresh Shetty, Saravanan Yuvaraja, Jose L. Cerrillo, Veerappan Mani, Khaled Nabil Salama**

Sensors Lab, Advanced Membranes and Porous Materials Center, Computer, Electrical and Mathematical Science and Engineering Division, King Abdullah University of Science and Technology (KAUST), Saudi Arabia.

ORCID IDs: G.T.B: 0000-0001-9045-613X; S.S.S.: 0000-0001-8905-4504; S.Y.: 0000-0001-9187-0543; J.L.C.: 0000-0001-8824-8294; V.M.: 0000-0002-0756-7398; K.N.S.: 0000-0001-7742-1282

[2]KAUST Catalysis Center (KCC), King Abdullah University of Science and Technology (KAUST), Saudi Arabia.

Corresponding author: veerappan.mani@kaust.edu.sa, khaled.salama@kaust.edu.sa

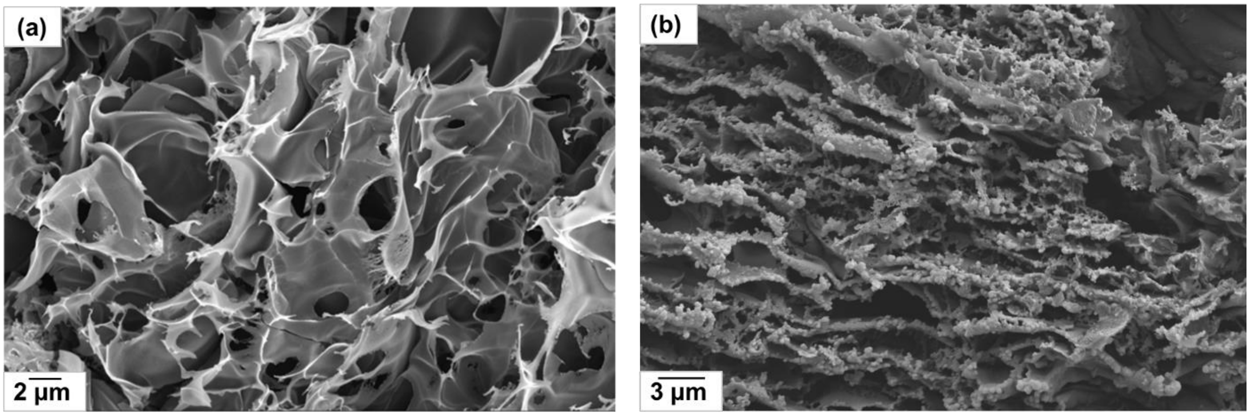

Figure S1. SEM images of LSG (a) and CuSAN (b).

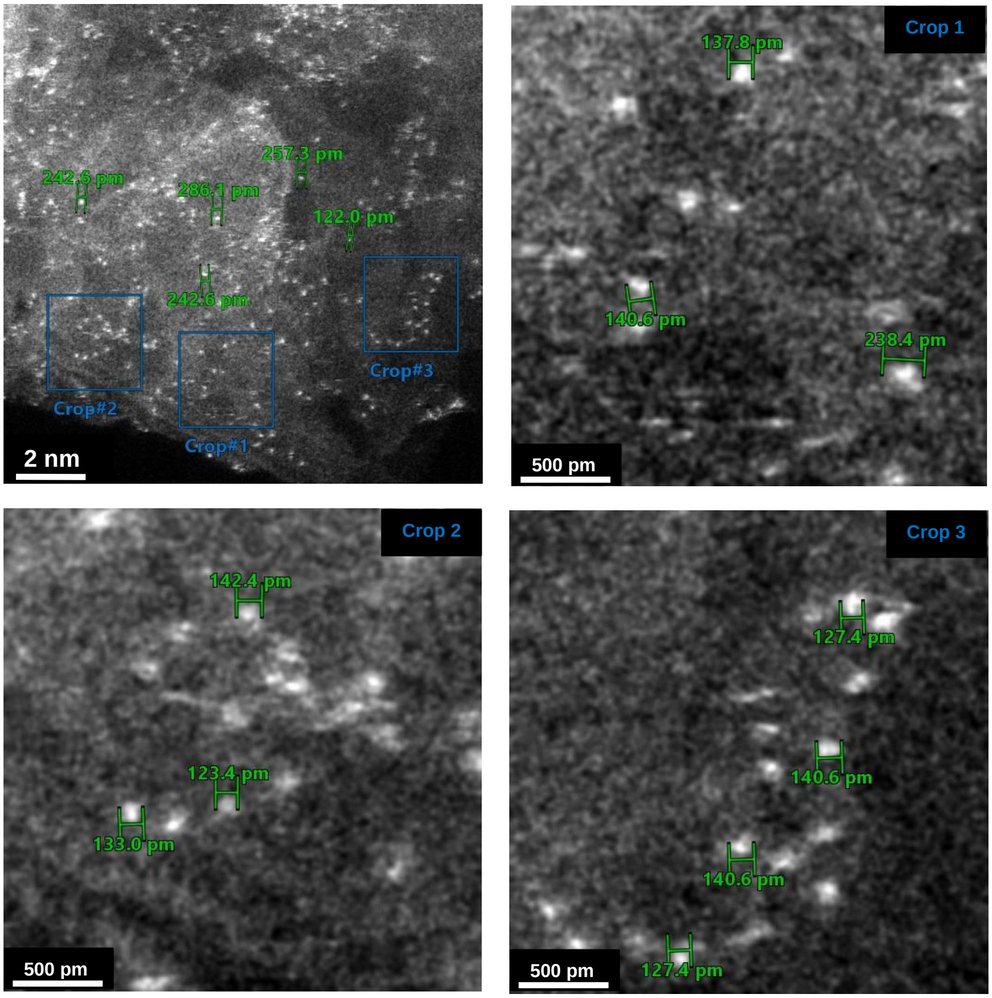

Figure S2. STEM images showcasing the size distribution of copper single atoms over the support.

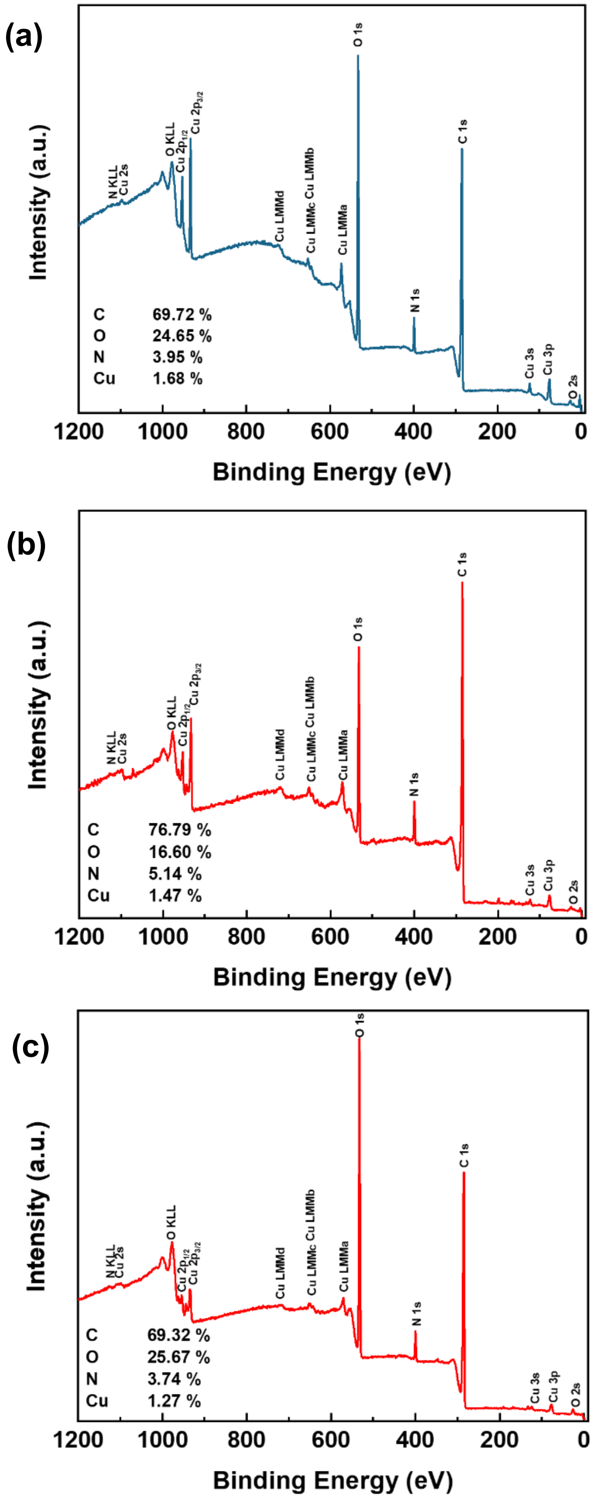

Figure S3. XPS Wide spectra of three different samples of CuSANs.

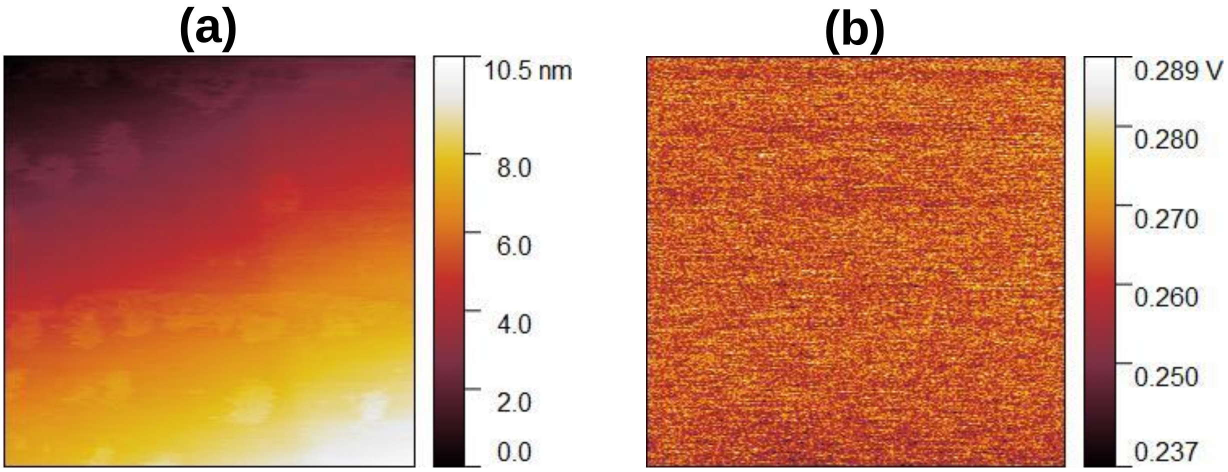

Figure S4. (a) Surface and (b) KPFM mapping of the standard HOPG sample

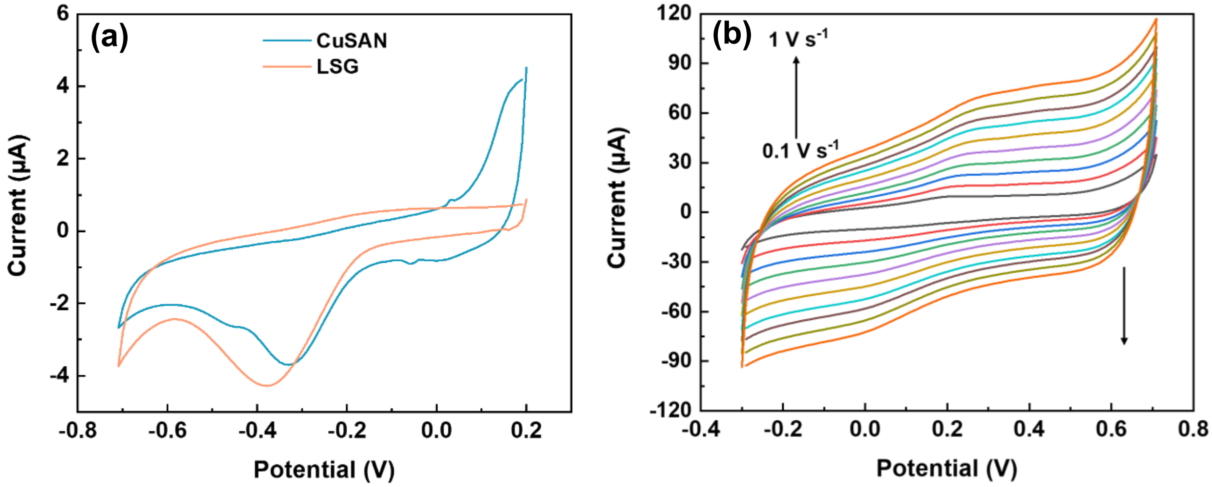

Figure S5. (a) CV response comparison of CuSAN and bare LSG electrodes in PBS, pH 7.40 at a scan rate of 10 mV s^{-1}. (b) CV responses at different scan rates for CuSAN electrode in PBS, pH 7.40, scan rate ranges from 0.1 V s^{-1} to 1.0 V s^{-1} where each curve represents a 0.1 V s^{-1} increment.

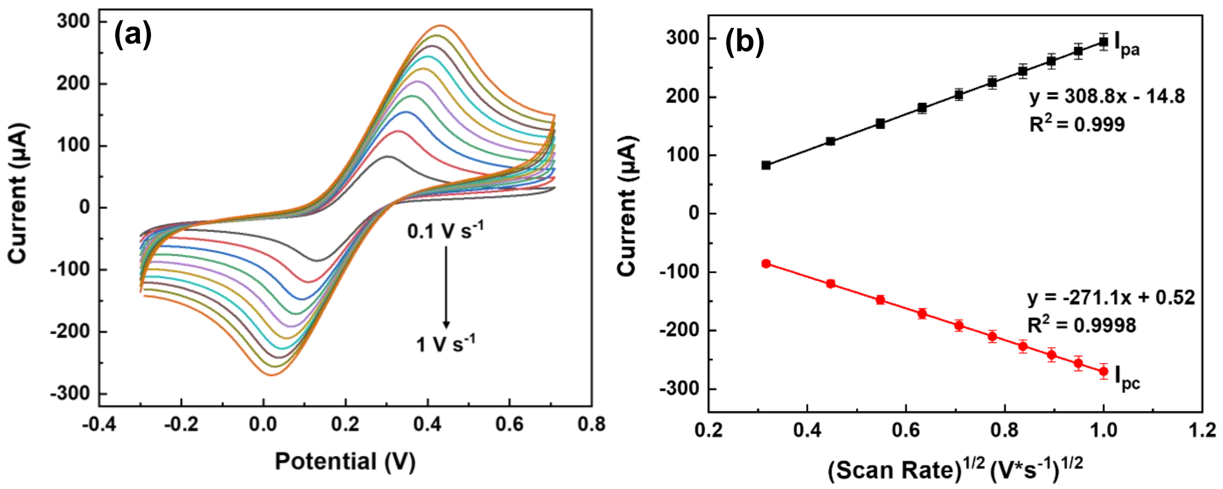

Figure S6. (a) CV responses of bare LSG electrode at different scan rates, for 5.0 mM $K_3Fe(CN)_6$ suspended in 0.1 M KCl. Scan rate ranges from 0.1 V s^{-1} to 1.0 V s^{-1} where each curve represents a 0.1 V s^{-1} increment. (b) Corresponding calibration plots between response current versus square root of scan rate. Slopes were employed to calculate the electrochemical active surface area (ECSA) of the LSG electrode.

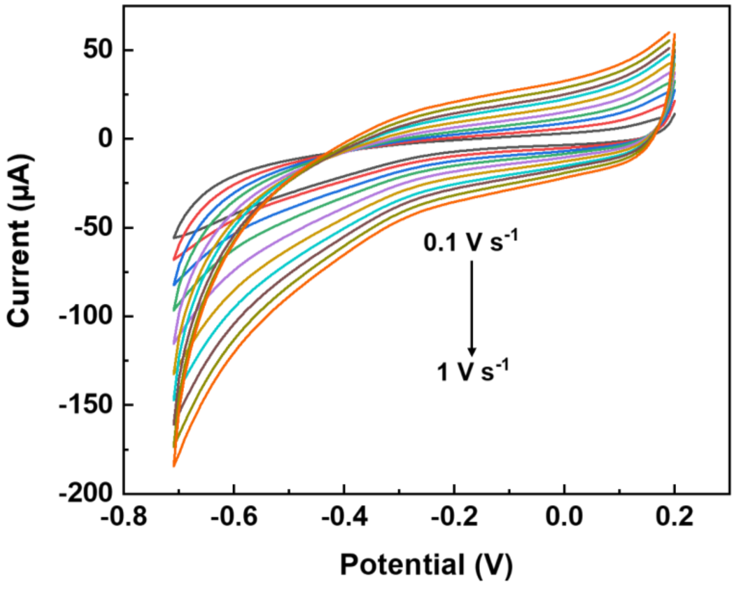

Figure S7. CV responses for 1.0 mM H_2O_2 at different scan rates using CuSAN electrode. Scan rate ranges from 0.1 V s^{-1} to 1.0 V s^{-1} where each curve represents a 0.1 V s^{-1} increment.

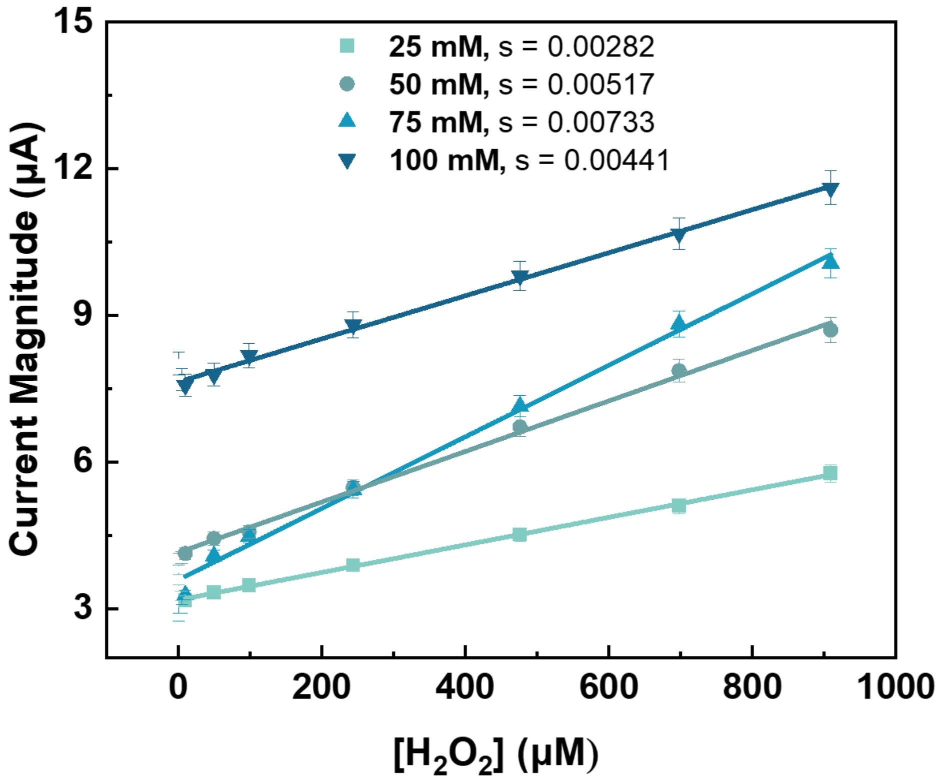

Figure S8. Calibration plots derived from the amperometric signals obtained with different copper loaded CuSAN. Different concentrations of H_2O_2 were tested in a range from 5 µM to 910 µM. Electrode potential: -0.38 V, supporting electrode was PBS, pH 7.40. (s) value indicates the slope for each system (µA μM^{-1}). Current magnitude stands for the background subtracted value of the measured current.

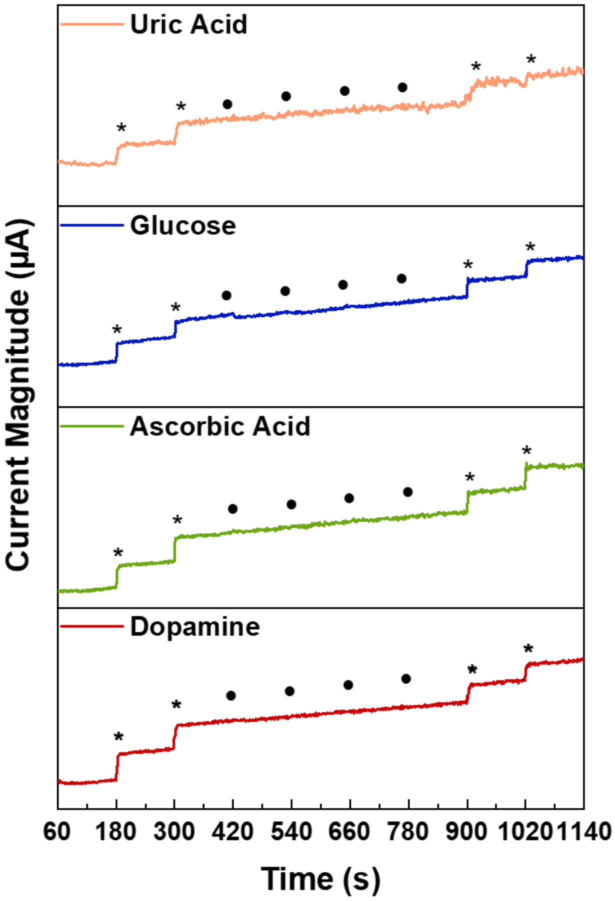

Figure S9. Amperometric signals of the CuSAN to different interferents at their biological range concentrations, where each (*) represent the addition of 250 μM H_2O_2 and each (•) represents a different concentration of interferent as follows: For DA (5.0, 10, 15 and 20 μM), for AA (25, 50 μM, 75 and 100 μM), for glucose (250, 500, 750 and 1000 μM) and for UA (50, 100, 150 and 200 μM).

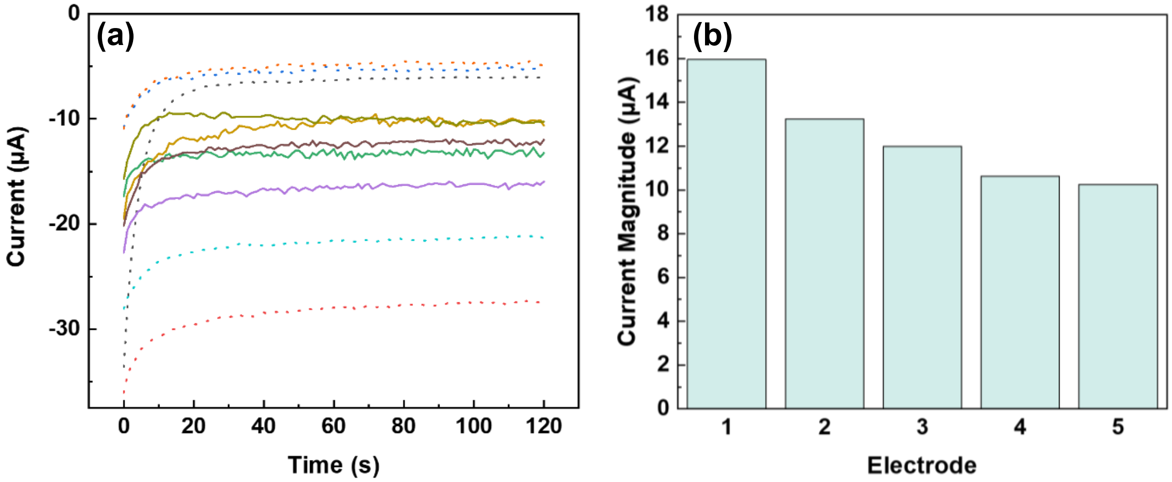

Figure S10. (a) Amperometric signal of different CuSAN working electrodes in PBS at a fixed potential of -0.38 V. The dotted lines represent the discriminated electrodes while the full lines represent the electrodes considered for the statistical analysis. (b) Bar chart comparing the five chosen electrodes closer in value to the average (12.69 V).

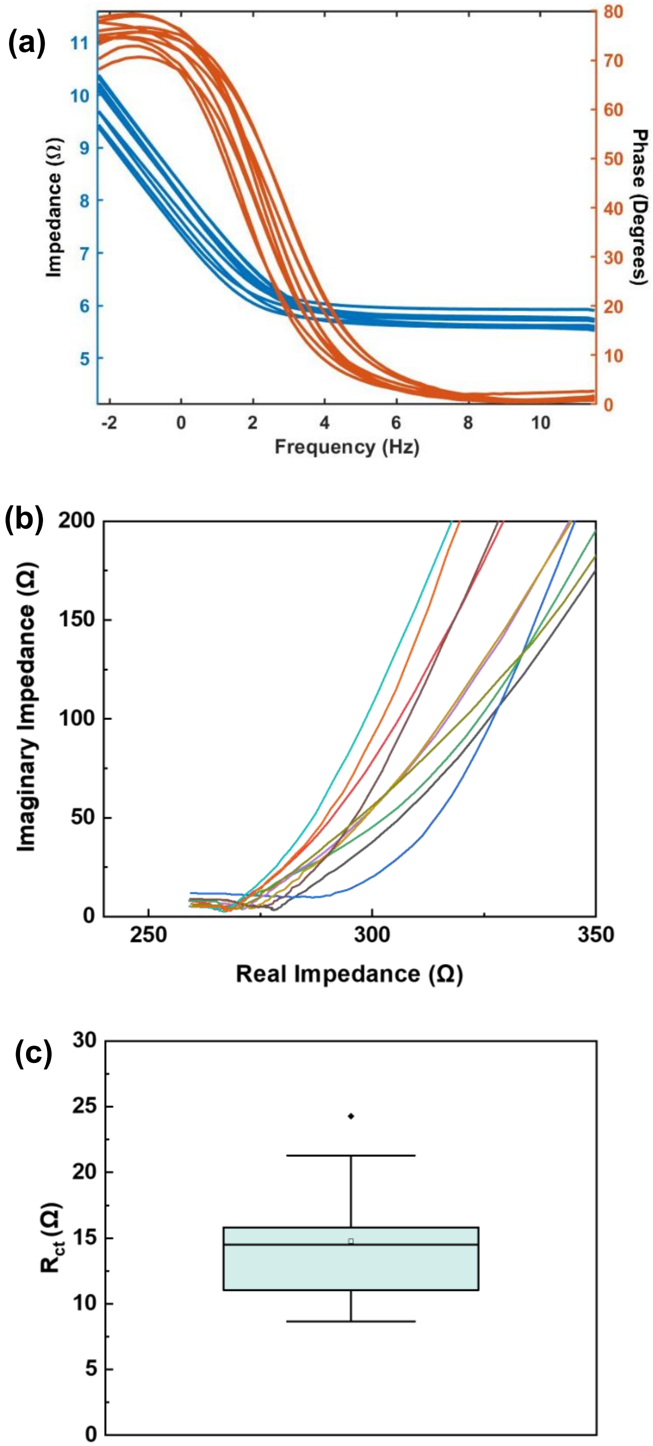

Figure S11. (a) Bode and (b)Nyquist plot corresponding to the impedance tests. c) Charge transfer resistance box plot derived from impedance measurements to justify the statistical exclusion of half of the population.

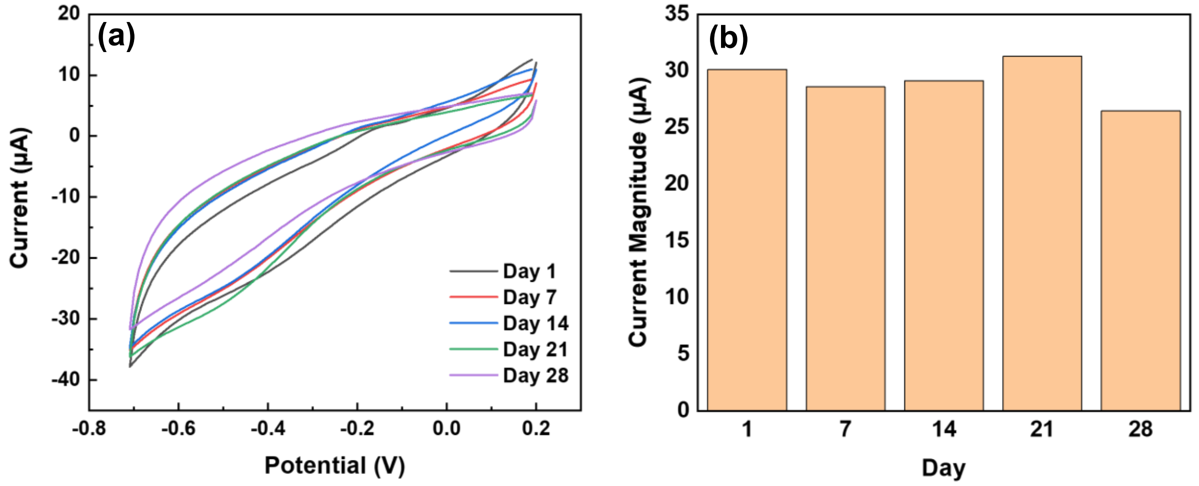

Figure S12. (a) CV responses of CuSANs/LSG electrode to 1.0 mM H_2O_2 over 28 days and (d) corresponding bar chart comparing the current signals. The supporting electrode was PBS, pH 7.40 and the scan rate was 50 mV/s

Table S1. A summary of the state-of-the-art applications of SANs

SAzyme	Active Site	Application
Oxidase [1]	Carbon nanoframe confined FeN	Wound Disinfection and Bactericidal.
Peroxidase and Oxidase [2]	Cu-N-C	Inhibitory effect on wound bacteria
Peroxidase [3]	Fe-N-C	In Situ colorimetric detection of H_2O_2 produced from Hela Cells.
Peroxidase [4]	Fe dispersed N-doped carbon/CNTs	Colorimetric detection of H_2O_2, glucose, and ascorbic acid.
Peroxidase [5]	Cu sites on N-doped Carbon Nanosheets	Colorimetric detection of acetylcholine and organophosphorus pesticides.
Peroxidase [6]	Pt supported on carbon nitride nanorod	Colorimetric antibiotic detection and antibacterial applications.
Peroxidase [7]	Fe sites coupled carbon encapsulated Fe_3C	Electrochemical monitoring of H_2O_2 released from living cells.
Laccase [8]	Fe anchored to N-doped carbon	Colorimetric detection and degradation of phenolic compounds.
Laccase [9]	Rh-N/C	Colorimetric detection of acetylcholinesterase activity and adrenaline.
Superoxide dismutase, Catalase [10]	Fe-N4 sites anchored on N-doped porous carbon	Cell protection by reactive oxygen species scavenging.

Table S2. Average copper mass loading of CuSANs

Sample	Average	Standard Deviation	Average	Standard Deviation
S1	0.17	0.01	0.19	0.02
S1 - Diluted	0.20	0.01		
S2	0.22	0.02	0.23	0.02
S2 - Diluted	0.24	0.01		
S3	0.27	0.02	0.29	0.02
S3 - Diluted	0.30	0.01		
S4	0.17	0.01	0.18	0.1
S4 - Diluted	0.19	0.01		
S4	0.17	0.01		
S4 - Diluted	0.19	0.01		

Table S3. Comparison of sensing analytical parameters of H_2O_2 with previous reports.

Electrode	Linear Range (mM)	LoD (μM)	Sensitivity (μA μM^{-1} cm^{-2})
Reduced graphene oxide/Ag nanoparticles [11]	0.002 - 20	0.73	236
Nickel-cobalt sulfide/rGO [12]	0.025 - 11.25	0.19	118.5
ZnO nanorods/vertical graphene nanowalls [13]	2.0 - 15	730	57.6
Prussian blue/3D printed graphene electrode [14]	0.001 - 0.7	0.11	86.9
Graphene nanosheets/FeOOH nanoparticles [15]	0.00025 - 1.2	0.08	265.7
Graphene/copper based nanoparticles [16]	0.032 - 0.803	0.64	0.37
Pt nanowires/peptide nanofibers/graphene oxide [17]	0.00005 - 15	0.0206	-
Nickel cobalt phosphide nanosheet/3D graphene [18]	0.0018 - 9.33	0.028	4398
Ag doped δ-MnO_2 nanorods/graphene [19]	0.005 - 90.64	0.068	104.43
CuSAN (This work)	0.003 - 0.91	2.087	130.0

Table S4. Comparison of sensing analytical parameters of H_2O_2 with other Cu-based nanozymes.

Electrode	Linear Range (mM)	LoD (μM)	Sensitivity (μA mM^{-1} cm^{-2})
N-doped carbon nanotubes anchored bimetallic cobalt copper organic frameworks [20]	0.05 - 3.5	0.206	639.5
Cu_2O/Electrochemically reduced graphene oxide [21]	0.001 - 1	0.14	168200
Copper/cuprous oxide nanocomposites [22]	0.0002 - 2	0.04	-
CuO nanosheets [23]	0.01 - 20	10	25.5
Copper nanoclusters [24]	0.01 - 1	10	-
Cu–metal organic frameworks [25]	0.025 - 30	25	263
Montmorillonite/CuS nanoparticles [26]	0.03 - 0.2	0.247	-
CuO flower-like nanostructured electrode [27]	0.425 – 0.04	0.167	88.4
CuSAN (This Work)	0.003 - 0.91	2.087	130000

References

[1] L. Huang, J. Chen, L. Gan, J. Wang, S. Dong, *Science Advances* **2019**, 5, eaav5490.
[2] J. Zhu, Q. Li, X. Li, X. Wu, T. Yuan, Y. Yang, *Langmuir* **2022**, 38, 6860.
[3] L. Jiao, W. Xu, H. Yan, Y. Wu, C. Liu, D. Du, Y. Lin, C. Zhu, *Analytical Chemistry* **2019**, 91, 11994.
[4] N. Cheng, J.-C. Li, D. Liu, Y. Lin, D. Du, *Small* **2019**, 15, 1901485.
[5] Y. Wu, J. Wu, L. Jiao, W. Xu, H. Wang, X. Wei, W. Gu, G. Ren, N. Zhang, Q. Zhang, L. Huang, L. Gu, C. Zhu, *Analytical Chemistry* **2020**, 92, 3373.
[6] Y. Fan, X. Gan, H. Zhao, Z. Zeng, W. You, X. Quan, *Chemical Engineering Journal* **2022**, 427, 131572.
[7] X. Wei, S. Song, W. Song, W. Xu, L. Jiao, X. Luo, N. Wu, H. Yan, X. Wang, W. Gu, L. Zheng, C. Zhu, *Analytical Chemistry* **2021**, 93, 5334.
[8] Y. Lin, F. Wang, J. Yu, X. Zhang, G.-P. Lu, *Journal of Hazardous Materials* **2022**, 425, 127763.
[9] J. Guan, M. Wang, R. Ma, Q. Liu, X. Sun, Y. Xiong, X. Chen, *Sensors and Actuators B: Chemical* **2023**, 375, 132972.
[10] W. Ma, J. Mao, X. Yang, C. Pan, W. Chen, M. Wang, P. Yu, L. Mao, Y. Li, *Chemical Communications* **2019**, 55, 159.
[11] P. Salazar, I. Fernández, M. C. Rodríguez, A. Hernández-Creus, J. L. González-Mora, *Journal of Electroanalytical Chemistry* **2019**, 855, 113638.
[12] M. Wang, J. Ma, X. Guan, W. Peng, X. Fan, G. Zhang, F. Zhang, Y. Li, *Journal of Alloys and Compounds* **2019**, 784, 827.
[13] T. Hang, S. Xiao, C. Yang, X. Li, C. Guo, G. He, B. Li, C. Yang, H.-j. Chen, F. Liu, S. Deng, Y. Zhang, X. Xie, *Sensors and Actuators B: Chemical* **2019**, 289, 15.
[14] V. Katic, P. L. dos Santos, M. F. dos Santos, B. M. Pires, H. C. Loureiro, A. P. Lima, J. C. M. Queiroz, R. Landers, R. A. A. Muñoz, J. A. Bonacin, *ACS Applied Materials & Interfaces* **2019**, 11, 35068.
[15] X. Chen, J. Gao, G. Zhao, C. Wu, *Sensors and Actuators B: Chemical* **2020**, 313, 128038.
[16] L. M. Alencar, A. W. B. N. Silva, M. A. G. Trindade, R. V. Salvatierra, C. A. Martins, V. H. R. Souza, *Sensors and Actuators B: Chemical* **2022**, 360, 131649.
[17] D. Zhu, P. He, H. Kong, G. Yang, X. Luan, G. Wei, *Journal of Materials Chemistry B* **2022**, 10, 9216.
[18] Y. Zhu, X. Ma, X. Lv, L. Zhang, C. Li, N. Shi, J. Wang, *Microchimica Acta* **2022**, 189, 345.
[19] A. K. Mohiuddin, S. Jeon, *Applied Surface Science* **2022**, 592, 153162.
[20] S. E. Kim, A. Muthurasu, *Electroanalysis* **2021**, 33, 1333.
[21] H. Ö. Doğan, E. Çepni, B. K. Urhan, M. Eryiğit, *ChemistrySelect* **2019**, 4, 8317.
[22] L. Han, L. Tang, D. Deng, H. He, M. Zhou, L. Luo, *Analyst* **2019**, 144, 685.
[23] Y.-K. Hsu, Y.-C. Chen, Y.-G. Lin, *Applied Surface Science* **2015**, 354, 85.
[24] L. Hu, Y. Yuan, L. Zhang, J. Zhao, S. Majeed, G. Xu, *Analytica Chimica Acta* **2013**, 762, 83.
[25] S. S. Menon, S. V. Chandran, A. Koyappayil, S. Berchmans, *ChemistrySelect* **2018**, 3, 8319.
[26] L. Zhang, M. Chen, Y. Jiang, M. Chen, Y. Ding, Q. Liu, *Sensors and Actuators B: Chemical* **2017**, 239, 28.
[27] M.-J. Song, S. W. Hwang, D. Whang, *Talanta* **2010**, 80, 1648.
